# Enhanced tumor control in non-small cell lung cancer using intensity-modulated radiotherapy combined with microwave hyperthermia: A retrospective study

**DOI:** 10.1097/MD.0000000000048529

**Published:** 2026-05-01

**Authors:** Wei Li, Xiangdong Liu, Wenyong Chen

**Affiliations:** a Department of Integrated Traditional Chinese and Western Medicine, Bijie Cancer Hospital, Bijie City, Guizhou Province, China; b Department of Oncology, Bijie Cancer Hospital, Bijie City, Guizhou Province, China.

**Keywords:** intensity-modulated radiotherapy, microwave hyperthermia, non-small cell lung cancer, tumor control

## Abstract

This study aimed to investigate the enhanced tumor control in patients with non-small cell lung cancer (NSCLC) using intensity-modulated radiotherapy (IMRT) combined with microwave hyperthermia (MWD). We retrospectively analyzed the clinical data of 134 patients with NSCLC admitted to our hospital from March 2021 to February 2024. Among them, 66 patients received IMRT alone (IMRT group) and 68 patients received IMRT combined with MWD (combined group). Tumor control rates, serum tumor markers, immune function, and toxic side effects were evaluated. The tumor control rate was significantly higher in the combined group (82.35%) than in the IMRT group (69.70%; *P* < .05). After treatment, the combined group showed significantly lower levels of carbohydrate antigen 125 (CA125), cytokeratin 19-fragments (Cyfra21-1), and carcinoembryonic antigen (CEA) compared to the IMRT group (*P* < .05). Furthermore, the levels of CD3+, CD4+, and CD4+/CD8+ ratios in the combined group were significantly higher than those in the IMRT group (*P* < .05). No significant difference in toxic side effects was observed between the 2 groups (*P* > .05). IMRT combined with MWD for NSCLC can effectively downregulate tumor markers, restore immune function, and improve tumor control rates without increasing toxicity.

## 1. Introduction

Lung cancer is the leading cause of cancer-related deaths worldwide.^[1]^ The main types of lung cancer are non-small cell lung cancer (NSCLC) and small cell lung cancer (SCLC), of which NSCLC accounts for 80% to 85% of lung cancer.^[2]^ In recent years, with the aggravation of environmental pollution, the incidence of NSCLC continues to increase, and the lack of specific clinical manifestations in the early stage of the disease, the majority of cases are already in the middle or late stages when diagnosed, losing the opportunity for surgical treatment.^[2,3]^ In such cases, comprehensive interventions, such as radiotherapy and chemotherapy, are needed to ensure quality of life and prolong life expectancy for patients.^[3,4]^

Intensity-modulated radiation therapy (IMRT) is a commonly used radiotherapy measure for NSCLC, which can maximize the radiotherapy dose to the target area and reduce the damage to the surrounding normal tissues and organs by optimizing the radiotherapy plan and system design to ensure therapeutic effect.^[5,6]^ Beyond NSCLC, IMRT has demonstrated significant advantages in various malignancies, such as head and neck cancers and prostate cancer, by providing superior dose conformity to complex target volumes while sparing adjacent organs at risk (OARs), such as the parotid glands and rectum.^[7]^ However, IMRT possesses certain limitations, including an increased volume of normal tissue receiving low-dose integral radiation – which may elevate the risk of secondary malignancies – and a high sensitivity to intra-fractional organ motion and anatomical changes, necessitating more complex treatment planning and higher costs.^[7]^

Microwave hyperthermia (MWD) is an established therapeutic modality for NSCLC, primarily because cancer cells exhibit lower thermal tolerance compared to normal cells. Clinically, MWD is extensively utilized not only for NSCLC but also for the local control of superficial tumors, palliative pain management for bone metastases, and as a physical sensitizer in multimodal therapies.^[8]^ The rationale for its combined use with radiotherapy lies in its modulation of the tumor microenvironment. Recent research has identified that hypoxic tumors rely on specific chemical substrates as fuel, which may be linked to invadopodium formation and increased invasiveness.^[9]^ Hypoxia is a primary driver of radio-resistance; by increasing local blood flow and lesion tissue oxygenation, MWD can effectively mitigate these hypoxic barriers and create a synergistic effect with radiotherapy.^[9-12]^

Although both IMRT and MWD have been shown to be important treatments for NSCLC, the literature on the combination of IMRT and MWD for NSCLC is limited. Therefore, this study retrospectively analyzed the clinical data of patients with NSCLC treated with IMRT combined with MWD in our hospital with the aim to clarify the impact of IMRT combined with MWD on the tumor control rate in patients with NSCLC.

## 2. Materials and methods

### 2.1. Patients

This study was conducted in accordance with the ethical standards of the 1964 Declaration of Helsinki and its later amendments. The study protocol was approved by the Ethics Review Board of Bijie Cancer Hospital (No. BJ2LYY-LL-SCSP-003; March 7, 2024). Given the retrospective nature of this study and the fact that all patient data were anonymized prior to analysis, the requirement for informed consent was waived by the Ethics Review Board of Bijie Cancer Hospital. We retrospectively analyzed the clinical data of 134 patients with NSCLC admitted to our hospital from March 2021 to February 2024. According to the treatment method, patients who received IMRT alone were included in the IMRT group, and patients treated with IMRT combined with MWD were included in the combined group. Patients were included if they diagnosed with NSCLC through pathological examination^[13]^; at disease stage III; received IMRT or IMRT combined with MWD treatment; had complete clinical data; had no history of chest surgery; and had Karnofsky Performance Scale (KPS) ≥ 60 points. Patients were excluded if they with other benign and malignant tumors and with severe infections and anemia.

### 2.2. Treatment procedures

In the IMRT group, patients were instructed to lie supine with crossed arms on top of the head, the position was properly fixed, the scanning reference points were marked with the assistance of a metal simulator, and when the field of view was fixed, the treatment was carried out through dynamic multi-leaf collimator and linear accelerator. The radiotherapy dose was 2.0 to 2.5 Gy, once a day, 5 times a week, for a total of 6 weeks of treatment. In the combined group, MWD was adopted on the basis of the IMRT group, patients were guided to take a comfortable position on the treatment bed. The microwave power was set to 27 Hz, with the center of the radiotherapy target area as the MWD center, and the surface of the skin of the heat transfer area as the temperature measurement point. The heat transfer time was 50 minutes, and the heat transfer temperature was between 42°C and 43°C. It was done twice a week, with an interval of approximately 48 to 72 hours between the 2 sessions. MWD should begin within 1 hour after radiotherapy and continued until the end of radiotherapy.

### 2.3. Information collection

Baseline data, including sex, age, body mass index (BMI), disease stage, tumor type, and smoking status. Tumor control rate: according to the evaluation criteria for the efficacy of solid tumors, it was divided into complete response, partial response, stability, and progression. Complete response, partial response, and stability were included in the tumor control rate.^[14]^ Serum tumor marker levels: fasting venous blood (4 mL) was extracted, centrifuged, and the supernatant was collected. The levels of carbohydrate antigen 125 (CA125) and cytokeratin 19-fragments (Cyfra21-1) were measured using double-antibody sandwich enzyme-linked immunosorbent assay; chemiluminescent immunoassay was used to determine the level of carcinoembryonic antigen (CEA). Immune function: fasting venous blood (4 mL) was extracted and CD3+, CD4+, and CD4+/CD8+ cells were measured using Beckman Coulter CytoFLEX flow cytometry. Toxic side effects include radiation pneumonitis, radiation-induced esophagitis, gastrointestinal reactions, and low white blood cell counts.

### 2.4. Statistical analysis

Statistical analysis was performed using SPSS version 26.0 (IBM Corp, Armonk). Continuous variables are expressed as mean ± standard deviation (SD) and were compared using independent-sample *t* tests between groups or paired *t* tests for within-group pre and posttreatment assessments. Categorical data are presented as frequencies and percentages, analyzed via the chi-square test. A *P*-value < .05 was considered statistically significant.

## 3. Results

A total of 134 patients (86 males and 48 females) met the inclusion and exclusion criteria were included in this study, including 66 cases in the IMRT group and 68 cases in the combined group. Patients aged from 43 to 78 years old (mean age, 60.37 ± 8.58 years). There was no significant difference in baseline data between the 2 groups of patients (*P* > .05; Table 1).

**Table 1:** Comparison of baseline data between 2 groups.

Baseline data	Combined group (n = 68)	IMRT group (n = 66)	*t*/χ^2^	*P*
Gender (male/female)	46/22	40/26	0.722	.395
Age (yr)	59.75 ± 8.18	61.02 ± 8.99	−0.852	.396
BMI (kg/m^2^)	23.47 ± 2.62	22.92 ± 3.11	1.108	.270
Disease staging (IIIa/IIIb)	35/33	38/28	0.503	.478
Pathological type				
Squamous cell carcinoma	37 (54.41)	33 (50.00)	0.699	.705
Adenocarcinoma	28 (41.18)	28 (42.42)		
Others	3 (4.41)	5 (7.58)		
Smoking situation (yes)	32 (47.06)	30 (45.45)	0.035	.852

Combined group refers to intensity-modulated radiotherapy combined with microwave hyperthermia, IMRT group refers to intensity-modulated radiotherapy.

BMI = body mass index.

The tumor control rate in the combined group (82.35%) was higher than that in the IMRT group (69.70%; *P* < .05; Table 2).

**Table 2:** Comparison of tumor control rates between 2 groups.

Group	n	Complete remission	Partial remission	Stable	Progression	Tumor control rate
Combined group	68	3 (4.41)	41 (60.29)	12 (17.65)	12 (17.65)	56 (82.35)
IMRT group	66	0 (0.00)	25 (37.88)	19 (28.79)	22 (33.33)	44 (66.67)
*χ^2^*						4.352
*P*						.037

Combined group refers to intensity-modulated radiotherapy combined with microwave hyperthermia, IMRT group refers to intensity-modulated radiotherapy.

**Figure 1.**
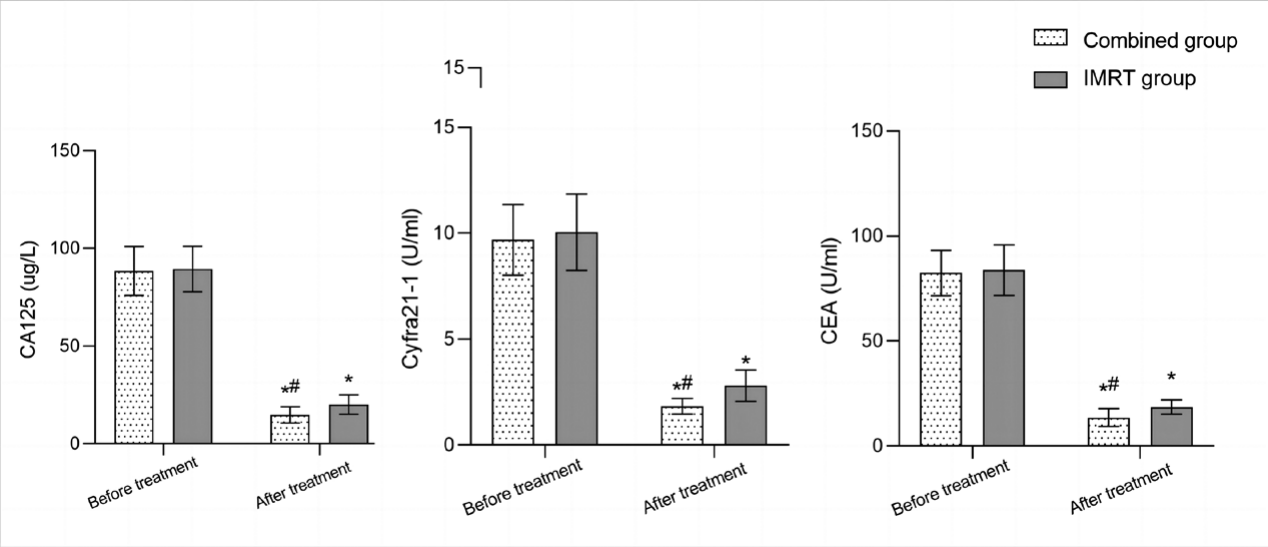
Comparison of serum tumor marker levels before and after treatment between 2 groups; comparison with before treatment in the same group, ^*^P < .05; compared to the IMRT group, ^#^*P* < .05; carbohydrate antigen 125 (CA125); soluble fragment of cytokeratin 19 (Cyfra21-1); carcinoembryonic antigen (CEA). CA125 = carbohydrate antigen 125, CEA = carcinoembryonic antigen, Cyfra21-1 = cytokeratin 19-fragments, IMRT = intensity-modulated radiotherapy.

Before treatment, there was no significant difference in serum CA125, Cyfra21-1, and CEA levels between the 2 groups (*P* > .05). After treatment, the serum levels of CA125, Cyfra21-1, and CEA in both groups significantly decreased compared to before treatment, and the combined group was significantly lower than the IMRT group (*P* < .05; Fig. 1).

**Figure 2.**
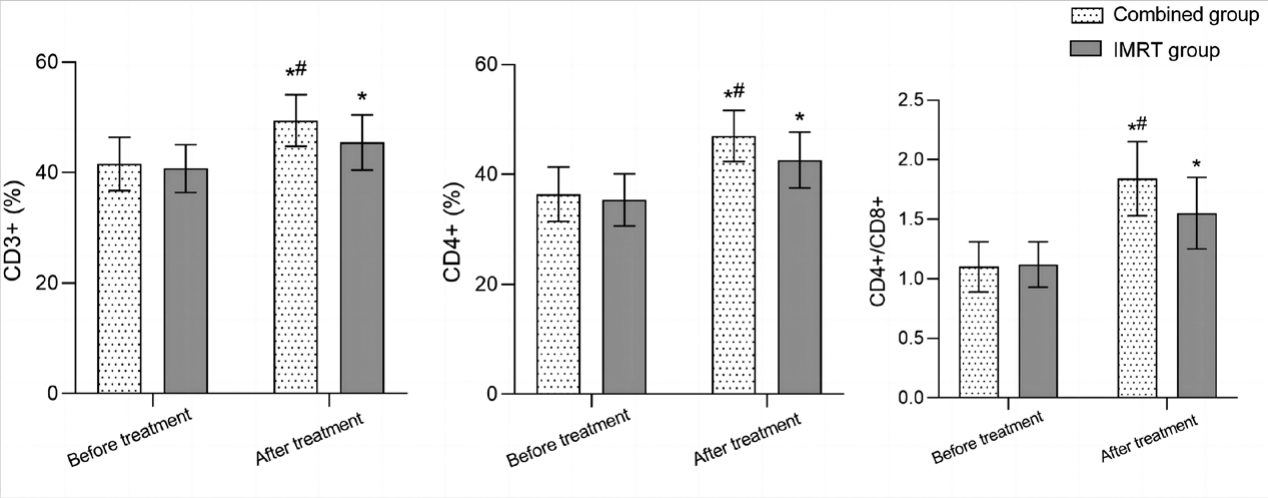
Comparison of immune function between 2 groups before and after treatment; comparison with before treatment in the same group, ^*^P < .05; compared to the IMRT group, ^#^*P* < .05. IMRT = intensity-modulated radiotherapy.

Before treatment, there was no significant difference in the levels of CD3+, CD4+, and CD4+/CD8+ between the 2 groups (*P* > .05). After treatment, the levels of CD3+, CD4+, and CD4+/CD8+ in both groups significantly increased compared to before treatment, and the combined group was significantly higher than the IMRT group (*P* < .05; Fig. 2).

There was no significant difference in the incidence of radiation pneumonitis, radiation-induced esophagitis, gastrointestinal reactions, and low white blood cell counts between the combined group (1.47%, 2.94%, 38.24%, 42.65%, respectively) and the IMRT group (4.55%, 6.06%, 34.85%, 40.91%, respectively; *P* > .05; Table 3).

**Table 3:** Comparison of incidence rates of toxic side effects between 2 groups.

Group	n	Radiation pneumonitis	Radiation-induced esophagitis	Gastrointestinal reactions	Low white blood cell counts
Combined group	68	1 (1.47)	2 (2.94)	26 (38.24)	29 (42.65)
IMRT group	66	3 (4.55)	4 (6.06)	23 (34.85)	27 (40.91)
*χ^2^*		0.289	0.207	0.166	0.042
*P*		.591	.649	.684	.838

Combined group refers to intensity-modulated radiotherapy combined with microwave hyperthermia, IMRT group refers to intensity-modulated radiotherapy.

## 4. Discussion

This study showed that the tumor control rate in the combined group was higher than that in the IMRT group (82.35% vs 69.70%), the levels of tumor marker and immune function indicators were better in the combined group than in the IMRT group (*P* < .05), and no significant difference in the incidence of radiation pneumonia, radiation esophagitis, gastrointestinal reactions, and low white blood cell count between the groups (*P* > .05). This indicates that the combination of IMRT and MWD in the treatment of NSCLC can more effectively reduce tumor marker levels, restore immune function, and have a positive significance in improving tumor control effectiveness. Moreover, it did not significantly increase the incidence of toxic side reactions, indicating a certain level of safety.

The therapeutic efficacy observed in the combined group likely stems from the unique thermal sensitivity of the tumor microenvironment. Disordered vasculature within tumor lesions facilitates selective heat accumulation, enabling the targeted destruction of malignant cells.^[15,16]^ Furthermore, hyperthermia directly induces cytotoxicity by promoting protein denaturation and inhibiting cellular DNA repair mechanisms.^[17,18]^ Meanwhile, MWD can increase the blood flow of the lesion tissue, improve tissue oxygen supply, and reduce the sensitivity of cells to radiotherapy in hypoxic and low-pH environments, thereby creating a synergistic mechanism with radiotherapy.^[19,20]^ Yang et al^[21]^ explored the application value of MWD in NSCLC and found that microwave ablation and radiotherapy were used to treat patients with stage III NSCLC who were difficult to undergo surgical treatment. The results showed no significant difference in survival benefits between the 2 groups, indicating that MWD can be a potential therapeutic measure for patients with NSCLC who missed the opportunity for surgical treatment, which is consistent with the results of the present study. Yang et al^[21]^ analyzed the mechanism of action of MWD and pointed out that hyperthermia can affect the blood flow perfusion status of capillaries, thereby regulating the distribution of drugs within tissues and improving the absorption and utilization rate of radiation and chemotherapy drugs in tumor lesions. Yeo^[22]^ studied the application value of hyperthermia combined with radiotherapy in NSCLC patients also achieved similar results to this study: after the combination of hyperthermia and radiation therapy, the patient’s survival status was good during the 18 month follow-up period, and no serious toxic side reactions occurred during the treatment period. Cheng et al^[23]^ used MWD to treat patients with recurrent NSCLC after radiotherapy, and the results showed a significant extension of their survival cycle and a significant reduction in lesion volume. This finding is consistent with the results of the present study. Cheng et al^[23]^ also pointed out that the recurrence rate of the disease after the second MWD decreased from 50% to 20%, but the study did not follow up the patient’s prognosis, which is one of the limitations of this study. Dobrodev et al^[24]^ found that MWD could inhibit DNA repair and P-glycoprotein expression, thereby enhancing the efficacy of radiotherapy and chemotherapy. It also has bone marrow protective effects, which can reduce the toxicity caused by radiotherapy and chemotherapy. The results confirm that the tumor control rate of patients with NSCLC treated with local hyperthermia on the basis of radiotherapy and chemotherapy can reach 93.0%, which is slightly higher than the 82.35% observed in the current study. There may be a correlation between the different disease stages and underlying conditions of the selected cases. Moreover, Dobrodev et al used MWD based on radiotherapy and chemotherapy, which is more commonly used than chemotherapy in this study.

Additionally, Wei et al^[25]^ found that MWD can prolong the progression-free survival and overall survival of patients with NSCLC to 14 months and 47.8 months, respectively, and the incidence of toxic side effect was only 55.7%, which was close to the incidence of toxic side effects in this study, further confirming the safety of MWD for NSCLC treatment. In addition, Yang et al^[26]^ indicated that the combination of conventional and MWD treatment for patients with advanced NSCLC can achieve an effective rate of 81.2%, which is close to 82.35% in this study. It has been confirmed that MWD can play a synergistic role in NSCLC and improve tumor control. Studies have also pointed out that M-phase cells are more sensitive to radiotherapy and chemotherapy, whereas S-phase cells are less sensitive. After radiotherapy and chemotherapy, M-phase tumor cells are extensively damaged, the cell cycle is redistributed, and most tumor cells are in the S-phase, and the implementation of hyperthermia treatment in this period can thermally kill S-phase cells.^[27,28]^ Moreover, hyperthermia can inhibit the repair of potentially lethal and sublethal damage to tumor cells, thereby ensuring the overall therapeutic effect.^[28]^

### 4.1. Limitations

This study has several limitations that must be acknowledged. First, this was a single-center retrospective study, which may have selection bias. Second, the rates of vascular stenosis and ultrasound indicators may be influenced by human or technical factors. Third, the patient’s prognosis was not followed up, and a longer follow-up time is needed to verify the results. Finally, the impact of the 2 methods on the long-term functional recovery of patients was not analyzed. Further prospective, multicenter analysis using regression models for comparisons of baseline characteristics and adverse events between groups, adjusting for potential confounding, presenting both crude and adjusted results, along with their uncertainties, is recommended in future research.

## 5. Conclusion

IMRT combined with MWD in the treatment of patients with NSCLC can downregulate tumor marker levels, improve immune function, and enhance tumor control rate without increasing the risk of toxic side reactions.

## Author contributions

**Conceptualization:** Xiangdong Liu.

**Data curation:** Xiangdong Liu.

**Formal analysis:** Wei Li, Xiangdong Liu.

**Funding acquisition:** Wei Li, Wenyong Chen.

**Software:** Wenyong Chen.

**Investigation:** Wei Li, Wenyong Chen.

**Writing – original draft:** Wei Li.

**Writing – review & editing:** Xiangdong Liu.
